# Physicochemical Properties of Starches in Proso (Non-Waxy and Waxy) and Foxtail Millets (Non-Waxy and Waxy)

**DOI:** 10.3390/molecules24091743

**Published:** 2019-05-05

**Authors:** Qinghua Yang, Weili Zhang, Jing Li, Xiangwei Gong, Baili Feng

**Affiliations:** College of Agronomy, Northwest A&F University, State Key Laboratory of Crop Stress Biology in Arid Areas, Yangling, Xianyang 712100, China; qinghuayang@nwafu.edu.cn (Q.Y.); zhang1415203881@163.com (W.Z.); lijing1993@nwafu.edu.cn (J.L.); gxw199308@163.com (X.G.)

**Keywords:** millet, starch, physicochemical properties, amylose

## Abstract

Proso and foxtail millets are widely cultivated due to their excellent resistance to biotic and abiotic stresses and high nutritional value. Starch is the most important component of millet kernels. Starches with different amylose contents have different physicochemical properties. In this study, starches in proso (non-waxy and waxy) and foxtail millets (non-waxy and waxy) were isolated and investigated. All the starch granules had regular polygonal round shapes and exhibited typical “Maltese crosses”. These four starches all showed bimodal size distribution. The waxy proso and foxtail millets had higher weight-average molar mass and branching degree and lower average chain length of amylopectin. These four starches all presented A-type crystallinity; however, the relative crystallinity of waxy proso and foxtail millets was higher. The two waxy millets had higher onset temperature, peak temperature, conclusion temperature, and gelatinization enthalpy. However, the two non-waxy millets had higher setback viscosity, peak time, and pasting temperature. The significantly different physicochemical properties of waxy and non-waxy millet starches resulted in their different functional properties.

## 1. Introduction

Millets, a group of small-seeded grains, are one of the oldest cereals that appeared 10,000 years ago, earlier than wheat and rice. The most widely cultivated millets are foxtail millet, proso millet, pearl millet, finger millet, and kodo millet [1]. In China, the proso (*Panicum miliaceum* L.) [2] and foxtail millets (*Setaria italica*) [3] are widely cultivated because they both have excellent drought tolerance. The shelled proso and foxtail millets are called “Huangmi” and “Minor millet”, respectively. They are preferred by many consumers due to their high nutritional value [4]. Therefore, research about proso and foxtail millets is particularly important. Starch, which can be divided into amylose and amylopectin, is one of the most important components of millet grains [1]. According to the content of amylose, millet can be divided into non-waxy (high amylose content) and waxy (low amylose content). Some studies have indicated that non-waxy and waxy millets have significant differences in physicochemical characteristics and cooking edibility [4]. Although starch is the main factor contributing to these differences, few studies have compared the starches in non-waxy and waxy millets.

The differences in molecular structure between amylose and amylopectin lead to their variances in physicochemical and functional properties. Therefore, starches with different amylose contents have specific applications in food and non-food industries. Some studies in maize have reported that starches with different amylose contents have different thermal behaviors and functional properties [5]. In addition, the starches of maize with different amylose contents respond differently to annealing and propionylation [6,7]. The study of barley starch indicated that amylose influences the crystalline regions [8]. Some studies have also investigated the effects of amylose on the physicochemical properties of rice [9] and wheat [10]. In genetic resources of millets, wide variations in amylose can be observed [11]. However, few studies have compared the starch physicochemical properties in non-waxy and waxy millets.

Accordingly, in this study, we obtained starches from proso (non-waxy and waxy) and foxtail millets (non-waxy and waxy) and investigated their granule morphology, molecular weight, chain length distribution of amylopectin, crystalline structure, physical properties of starches, thermal properties, and pasting properties. Our results will provide a deeper understanding of the differences in waxy and non-waxy millet starches.

## 2. Results and Discussion

The chemical composition of flours and starches are summarized in Table 1. The contents of fat, protein, and starch in flours were 2.83–4.54%, 8.83–11.09%, and 71.79–76.43%, respectively. The waxy foxtail millet had the highest fat content, and the non-waxy proso millet had the lowest fat content. The proso millet had higher protein content than the foxtail millet. The high starch content in these four flours indicated that the proso and foxtail millets were good starch resources. The contents of fat, protein, and starch in starches were 0.01–0.02%, 0.80–1.30%, and 83.03–94.60%, respectively. The fat and protein contents in starches were both very low, which indicated that the isolated starches were pure. The amylose contents of flours in non-waxy proso millet, waxy proso millet, non-foxtail millet, and foxtail millet were 28.00%, 2.00%, 29.10%, and 2.00%, respectively. The amylose contents of starches in non-waxy proso millet, waxy proso millet, non-foxtail millet, and foxtail millet were 32.80%, 2.80%, 36.70%, and 2.50%, respectively. These results indicated that the flours and starches of non-waxy proso and foxtail millets had higher amylose content than those of waxy proso and foxtail millets.

### 2.1. Morphology and Size of Starch

**Figure 1:**
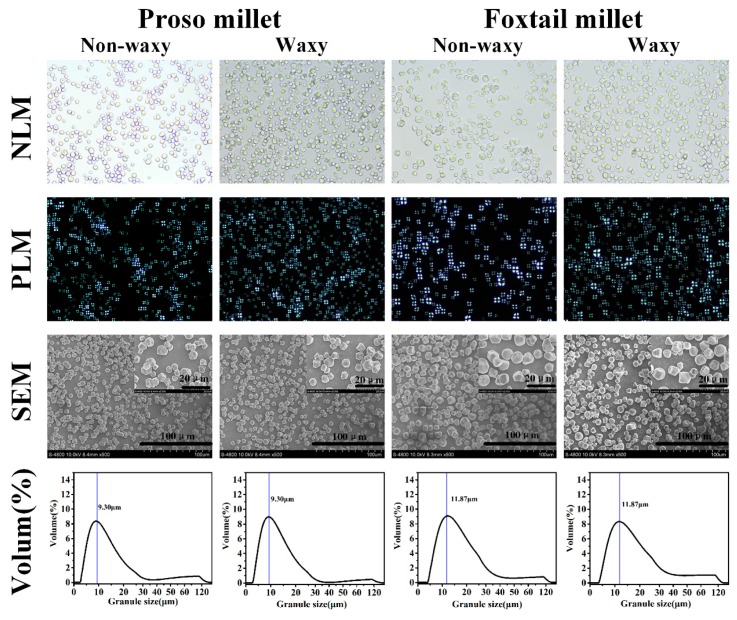
Morphologies of starch granules under normal light microscope (NLM), polarized light microscope (PLM), and scanning electron microscope (SEM), and the granule size distribution of starches.

The image of scanning electron microscope and polarized light exhibited that the starches all showed regular polygonal, round, and typical “Maltese crosses” (Figure 1), which were consistent with the results of previous studies [1]. Our results indicated that there was no significant difference in the morphology of waxy and non-waxy millets (proso and foxtail millets). These four starches had a bimodal size distribution, which indicated that they have a wide range of particle size distribution. The peak diameters, d (0.1), d (0.5) and d (0.9) values, of foxtail millet were significantly larger than those of proso millet (Figure 1, Table 2), indicating that the starch granules of foxtail millet were larger than those of proso millet. In this study, the d (0.9) value of non-waxy proso millet was larger than that of waxy proso millet, whereas the d (0.9) value of non-waxy foxtail millet was smaller than that of waxy foxtail millet. The results showed that although the waxy and non-waxy millets had the same peak diameter, the distribution of starch granules was different. However, Chao et al. reported that the average starch granule sizes of waxy proso millet were smaller than those of non-waxy proso millet [2], which may be due to different varieties or measurement methods.

**Figure 2:**
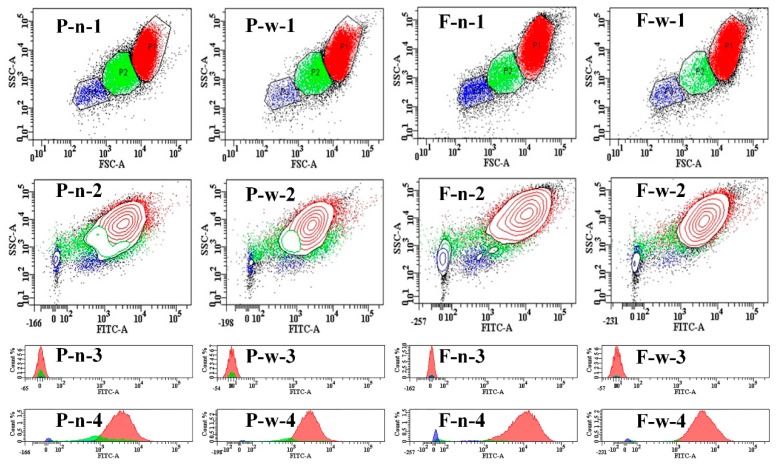
(P-n), non-waxy proso millet; (P-w), waxy proso millet; (F-n), non-waxy foxtail millet; (F-w), waxy foxtail millet. (1), forward-scattered light (FSC)-side-scattered light (SSC) image; (2), fluorescence image; (3), histogram of unstained starch (negative control); (4), histogram of 1-aminopyrene-3,6,8-trisulfonic acid (APTS)-stained starch.

Currently, flow cytometry is widely considered as an effective method for the classification of granules. So far, this method has only been used in corn starch [12]. Side-scattered light (SSC), forward-scattered light (FSC), and 1-aminopyrene-3,6,8-trisulfonic acid (APTS) represent the integral structure complexity, granule size, and fluorescence intensity (Figure 2), respectively. Moreover, the histogram of unstained and APTS-stained starches verified the authenticity of the results. P1 to P3 means an increase in the size and complexity of starch granules. The P3 of non-waxy proso millet was higher than that of waxy proso millet, whereas the P3 of non-waxy foxtail millet was lower than that of waxy foxtail millet. This result indicates that the starch granules of non-waxy proso and waxy foxtail millets were more complex and had wider distribution, which was consistent with the Mastersizer results. Compared with amylose, genotypes and growth conditions may have a greater impact on starch granule size [1].

### 2.2. Molecular Weight of Starch and Chain Length Distribution of Amylopectin

The branching degree and weight-average molar mass (*Mw*) of starches are presented in Table 3. The waxy proso (4.2%) and foxtail millets (3.9%) had higher branching degree than the non-waxy proso (2.8%) and foxtail millets (2.8%). Amylose was linked by α-1,4-glycosidic bond. However, amylopectin was mainly linked by α-1,6-glycosidic bond and a small part was mainly linked by α-1,4-glycosidic bond [13]. Therefore, the branching degree of starches was negatively correlated with the amylose. The *Mw* of non-waxy and waxy proso millets were 2.4 × 10^7^ and 17.0 × 10^7^ g/mol, respectively. The *Mw* of non-waxy and waxy foxtail millet were 2.8 × 10^7^ g/mol and 22.3 × 10^7^ g/mol, respectively (Table 3). Some studies reported that the *Mw* of amylopectin is higher than that of amylose [14]. The waxy millets had more amylopectin contents than non-waxy millets; therefore, the waxy proso and foxtail millets had higher *Mw*. Li et al. have reported that rice starch with high amylose tends to have smaller amylopectin and amylose molecular sizes [15]. In this study, a similar result was obtained in proso and foxtail millets.

**Figure 3:**
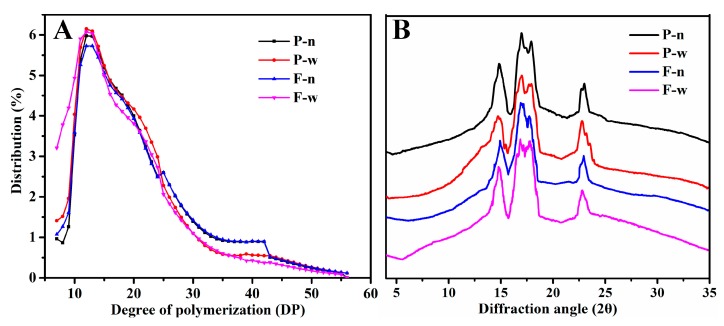
Amylopectin chain length distribution (**A**) and X-ray diffraction (XRD) patterns (**B**) of starches.

The amylopectin chain length distribution of starches notably influenced the crystalline structure, gel characteristics, and pasting properties of starches [16]. The branch chain length distribution of amylopectin is presented in Table 3 and Figure 3A. Amylopectin branch chains are generally classified into the following types according to their degree of polymerization (DP): B1 chain, B2 chain, and B3+chains. The average chain length of amylopectin in non-waxy proso (20.1) and foxtail millets (20.2) was higher than that of waxy proso (19.2) and foxtail millets (17.9). Compared with non-waxy proso millet, waxy proso millet had higher proportions of DP 6–12 and DP 13–24 but a lower amount of DP 25–37 and DP ≥37. In addition, waxy proso millet had higher proportions of DP 6–12, whereas non-waxy proso millet had higher proportions of DP 25–37 and DP ≥37. This result indicated that the proso and foxtail millets with high amylose had higher proportion of short branch chains and lower proportion of long branch chains, which was agreement with the studies in rice and maize [17].

### 2.3. Crystalline Structure

The four starches all presented an A-type diffraction pattern having double peaks approximately at 17° and 18° 2θ and two single peaks approximately at 15° and 23° 2θ. According to previous reports, most cereal starches present an A-type diffraction pattern. The same results were also obtained in proso and foxtail millets. Genkina et al. have reported that the crystallisation type of starch is not influenced by amylose, but the relative crystallinity is [18]. The relative crystallinities of non-waxy proso millet, waxy proso millet, non-waxy foxtail millet, and waxy foxtail millet were 37.6%, 38.4%, 34.1%, and 36.5%, respectively. Such differences in relative crystallinity can likely be attributed to the biological origin, crop variety, amylose and amylopectin content, growth conditions, and maturity of parent plant at harvest time. The relative crystallinity differed between non-waxy and waxy millets and this probably could be attributed to amylopectin chain length [2]. Amylopectin with a chain length of DP 13–24 is more likely to form the crystalline structure of starch. The waxy proso millet had the highest relative crystallinity, which may be due to its high proportion of DP 13–24. The waxy proso and foxtail millets had higher amylopectin contents than the non-waxy proso and foxtail millets. Thus, the proportion of DP 13–24 was higher in waxy proso and foxtail millets. This may be the reason why the waxy proso and foxtail millets had higher relative crystallinity than the non-waxy proso and foxtail millets.

### 2.4. Physical Properties of Starches

**Figure 4:**
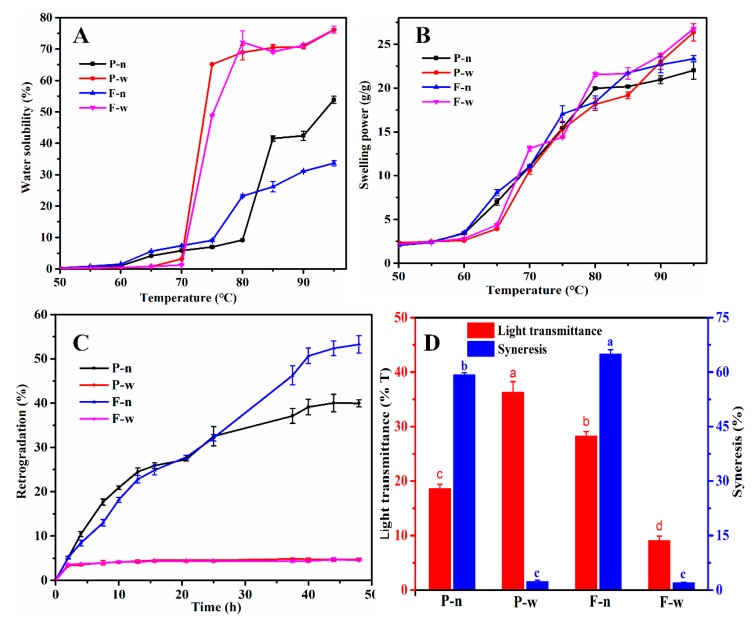
Physical properties of starches. (**A**), Water solubility of starch; (**B**), swelling power of starch; (**C**), retrogradation of starch; (**D**), light transmittance and freeze–thaw stability of starch.

The physical properties of starches, including water solubility, swelling power, retrogradation, light transmittance, and freeze–thaw stability, are vital indicators of starch quality and can provide a basis for the processing and utilization of starch. Figure 4A,B show the water solubility and swelling power of starches. The water solubility of starches in waxy proso and foxtail millets increased rapidly at 70–80 °C and increased slowly after 80 °C. However, the water solubility of starches in non-waxy proso and foxtail millets increased at a relatively steady speed after 60 °C. The swelling power of the four starches showed a similar trend with temperature changes, which increased at a relatively steady speed after 60 °C. The water solubility and swelling power of waxy proso and foxtail millets were higher than those of non-waxy proso and foxtail millets. Starch swelling and solubility occur with disruption of the structure caused by the breaking of hydrogen bonds between water molecules and the exposed hydroxyl group of amylose and amylopectin [5]. In addition, the water solubility and swelling power were affected by many factors (including amylose, amylopectin, and granule size) [19], and the differences between waxy and non-waxy millets may be due to the amylose content. Amylose inhibits the water solubility and swelling power of starches.

The retrogradation of two waxy millets increased during the first 2 h and then stabilized. However, the retrogradation of non-waxy millets increased during the first 40 h and then stabilized (Figure 4C). The retrogradation reflected the stability of starches, and the higher the retrogradation, the poorer the stability of starch [20]. These results showed that the retrogradation of waxy proso and foxtail millets was greatly lower than that of non-waxy proso and foxtail millets, which indicated that the starches of these two waxy millets had better stability. Starch retrogradation is a key factor limiting the applications of starch, which affects their nutritional values and sensory qualities. So the starches of waxy millets are good raw material resources for making drinks due to their low retrogradation [2].

The light transmittance of starch is one of the important indicators of starch paste, and starch products with good light transmittance are more popular amongst consumers. Figure 4D shows that the waxy proso and non-waxy foxtail millets had higher light transmittance. The better the distribution of starch granules, the better the transparency of starch. Based on the flow cytometry results, the starch granule distribution of waxy proso and non-waxy foxtail millets was more concentrated, which may be the reason why these two samples had better light transmittance. Freeze–thaw stability is an important property that is used to evaluate the ability of starch to withstand undesirable physical changes occurring during freezing and thawing [13]. The starch of waxy proso and foxtail millets had lower syneresis, indicating that these two starch samples had better freeze–thaw stability. The result was consistent with the retrogradation rate.

### 2.5. Thermal Properties of Starch

The thermal properties of starches are presented in Table 4. The onset temperature (To), peak temperature (Tp), conclusion temperature (Tc), and gelatinization enthalpy (ΔH) of starches were 64.6–71.1 °C, 70.5–77.9 °C, 76.3–82.3 °C, and 6.6–10.8 J/g, respectively. High gelatinisation temperature indicated a more perfect crystal structure in starch, and high ΔH reflected that more energy is required for starch gelatinisation [21]. The thermal properties of starch are related to many factors, including the morphology of starch granules, chain length distribution of amylopectin, and crystalline structure [22]. The waxy proso and foxtail millets had higher To, Tp, Tc, and ΔH than the non-waxy proso and foxtail millets, which indicated that waxy proso and foxtail millets have more stable and perfect structure. This result may also be related to their high proportions of DP 13–24 amylopectin and high relative crystallinity [23]. Amylopectin with a DP 13-24 is more likely to form crystalline structures, which is more difficult to be destroyed [23]. The proso millet starches had higher ΔH than foxtail millet, probably because starch granules in proso millet were smaller than starch granules in foxtail millet [13].

### 2.6. Pasting Properties of Starch

The pasting properties of starches measured by rapid visco analyzer (RVA) are presented in Table 4. The pasting temperature (PTM) and peak time (PT) of non-waxy proso millet, waxy proso millet, non-waxy foxtail millet, and waxy foxtail millet were 80.9 °C, 77.8 °C, 77.8 °C, and 76.5 °C and 4.5 min, 4.2 min, 4.3 min, and 4.1 min, respectively. The non-waxy proso and foxtail millets had higher PTM and PT, which indicated that they were more difficult to be gelatinized [24]. The lower setback viscosity (SB) in waxy proso and foxtail millets indicated that they had better stability, and this result was consistent with the retrogradation and syneresis of starch [22]. The lower breakdown viscosity (BD) in non-waxy proso and foxtail millets indicated that they had strong shear resistance [24]. In addition, the waxy proso and foxtail millets had higher peak viscosity (PV) and trough viscosity (TV). In this study, the waxy proso and foxtail millets had higher PV, TV, and BD, and lower SB and PT than the non-waxy millets. A study in proso millet has reported that the amylose content is highly negatively correlated with the PV, TV, and BD. Furthermore, the amylose content is highly positively correlated with the SB and PT [2], which was consistent with our results. These findings indicated that starches of waxy proso and foxtail millets had better stability and were suitable as thickeners and frozen convenience food and that starches of non-waxy proso and foxtail millets had better antishear ability and higher temperature resistance and were suitable for new materials in medicine.

## 3. Materials and Methods

### 3.1. Materials

The grains of non-waxy proso millet (Longmi 5), waxy proso millet (Yushu 1), non-waxy foxtail millet (Chigu 4) and waxy foxtail millet (Fente 5) were studied. These four varieties were planted at Yulin Modern Agriculture Demonstration Garden of Yulin, Shaanxi Province, China in 2018. The shelled seeds were turned into flour using a high-speed pulverizer (FW-100D, XinBoDe Instruments Ltd., Tianjin, China). Starches were isolated using the alkaline steeping method according to the description of Chao et al. [2].

### 3.2. Chemical Composition of Flour and Starch

The fat was obtained by Soxhlet extraction and the solvent is petroleum ether (boiling point 60–90 °C). The protein was obtained by Kjeldahl method and the content of protein (%) was calculated from the nitrogen content (N × 6.25). The total starch contents were obtained by anthrone spectrophotometric method and were calculated by multiplying glucose concentration by a conversion factor of 0.9, respectively [4]. The amylose content was measured following the method described by Yang et al. [4].

### 3.3. Morphology Observation of Starch

Starch was suspended (10% *w*/*v*) in 50% glycerol and then observed under normal and polarized light using BX53 (Olympus, Tokyo, Japan). The surface structures of starch were obtained using a scanning electron microscope (S4800, Hitachi Instruments Ltd., Tokyo, Japan) according to the method described by Chao et al. [2].

### 3.4. Granule Size Analysis of Starch

The distribution of granule size was determined using Mastersizer 2000 [25]. The complexity and size of starch granule were obtained using flow cytometry (BD FACSAria™ III, Franklin Lakes, NJ, USA), according to the method of Zhang et al. [12] with some modifications (the suspension was replaced with double distilled water and the unstained starch granules were used as negative control).

### 3.5. Branching Degree of Starch

The branching degree of starch was measured using the AVANCE III 400 MHz NMR Spectrometer (Bruker, karlsruhe, Switzerland) according to the method of Boonna [15].

### 3.6. Molecular Weight Distribution of Starch

*Mw* was analyzed using the Agilent PL-GPC 220 high-temperature chromatograph and multiangle laser-light scattering [26] with differential refractive index detector [27].

### 3.7. Chain Length Distribution of Amylopectin

Amylopectin branch chain length distribution of starch was measured using high-performance anion exchange chromatography [28]. Flow rate was set as 0.5 mL/min. The elution gradient was made with 500 mM sodium acetate in 150 mM NaOH against 150 mM NaOH as follows: 0–20% for 0–5 min, 20–45% for 5–15 min, 45–60% for 15–40 min, 60–70% for 40–65 min, and 70–100% for 75–80 min.

### 3.8. Crystalline Structure

According to the method described by Gao et al. [24], the crystalline structure of starch was analyzed with X-ray diffraction (XRD) (D8, Bruker, karlsruhe, Germany).

### 3.9. Physical Properties of Starch

The swelling power and water solubility of the starch were measured from 50 to 95 °C by increments of 5 °C according to the method described by Uarrota et al. [19].

Next, 0.25 g starch and 25 mL H_2_O were placed in a graduated glass test tube and then 100 °C water bath for 15 min. Finally, the tube was placed at 30 °C, the volume of the supernatant was recorded every 2 h, and the retrogradation was the change of supernatant volume percentage with time. The following formulae were used:

Retrogradation (%) = (Supernatant volume/Total volume) × 100(1)

Approximately 0.1 g starch and 10 mL H_2_O were placed in a test tube and shaken and then 100 °C water bath for 20 min. Finally, the solution was cooled at room temperature for 30 min, and then the light transmittances were obtained at 620 nm using a spectrophotometer.

The freeze–thaw stability of starch was measured using the method of Arunyanart and Charoenrein [29].

### 3.10. Thermal Property Analysis of Starch

Approximately 3 g starch and 6 mL H_2_O were mixed in an aluminum pan, sealed and then placed at room temperature for 2 h. The thermal property of starch was analyzed using DSC (Q 2000, TA Instruments Inc, Newcastle, DE, USA), whose program was set to heat up to 110 °C at a rate of 10 °C/min.

### 3.11. Pasting Property Analysis of Starch

Pasting properties were obtained using RVA 4500 (Perten, Sweden). Three grams of dried starch and 25 mL H2O were mixed, and then the mixture was heated to 50 °C and kept for 1 min. Next, the temperature was raised to 95 °C at a rate of 12 °C/min for 2 min. Finally, the temperature was cooled down to 50 °C at a rate of 12 °C/min and kept for 1 min. The sample was firstly dispersed for 10 s at a speed of 960 rpm and then a speed of 160 rpm was used throughout.

### 3.12. Statistical Analysis

All determinations were replicated three times and the data were reported as mean values ± standard deviations. Data were subjected to one-way analysis of variance and Tukey’s multiple-comparison analysis using SPSS 16.0 (SPSS Inc., Chicago, IL, USA).

## 4. Conclusions

Starches were isolated from proso (non-waxy and waxy) and foxtail millets (non-waxy and waxy). The granules of all starches had regular polygonal round shapes and showed typical “Maltese crosses”; however, they exhibited different granule size distribution. The waxy proso and foxtail millets had higher *Mw* and branching degree. The proso and foxtail millets with high amylose had higher proportion of short branch chains and lower long branch chains. These four starches all presented A-type crystallinity; however, the relative crystallinities of waxy proso and waxy foxtail millets were higher. The four starch pastes had differences in water solubility, swelling power, retrogradation, light transmittance, and syneresis. The starches of waxy proso and non-waxy foxtail millets had better light transmittance. The waxy proso and foxtail millets required more energy for starch gelatinization. Starches of waxy proso and foxtail millets had better stability. Starches of non-waxy proso and foxtail millets had better anti-shear ability and higher temperature resistance. The above results could provide important information for the utilization of starches with different amylose contents from proso and foxtail millets.

## Figures and Tables

**Table 1:** Chemical compositions of flours and starches.

Varieties	Flour				Starch			
	Fat Content (%)	Protein Content (%)	Starch Content (%)	Amylose Content (%)	Fat Content (%)	Protein Content (%)	Starch Content (%)	Amylose Content (%)
P-n	2.83 ± 0.12 ^c^	11.04 ± 0.05 ^a^	76.43 ± 1.21 ^a^	28.00 ± 0.70 ^a^	0.01 ± 0.01 ^a^	1.30 ± 0.06 ^a^	87.23 ± 0.56 ^b^	32.80 ± 0.70 ^b^
P-w	4.37 ± 0.20 ^b^	11.09 ± 0.09 ^a^	75.37 ± 0.94 ^a^	2.00 ± 0.20 ^b^	0.01 ± 0.00 ^a^	1.07 ± 0.26 ^a b^	94.60 ± 1.30 ^a^	2.80 ± 0.20 ^c^
F-n	3.85 ± 0.21 ^b^	8.83 ± 0.05 ^c^	71.79 ± 0.92 ^b^	29.10 ± 1.30 ^a^	0.02 ± 0.01 ^a^	0.89 ± 0.06 ^b^	87.15 ± 0.96 ^b^	36.70 ± 0.40 ^a^
F-w	4.54 ± 0.50 ^a^	10.07 ± 0.19 ^b^	72.04 ± 0.93 ^b^	2.00 ± 0.30 ^b^	0.01 ± 0.01 ^a^	0.80 ± 0.18 ^b^	83.03 ± 1.31 ^c^	2.50 ± 0.10 ^c^

Data represent means ± standard deviations. For each column, values not displaying the same letter are significantly different (*p* < 0.05).

**Table 2:** Granule distribution of starches.

Varieties	Granule Size (μm)					Particle Percentage of Subgroups in Different Starches (%)		
	d (0.1)	d (0.5)	d (0.9)	D (3, 2)	D (4, 3)	P1	P2	P3
P-n	4.49 ± 0.01 ^d^	8.93 ± 0.08 ^b^	22.47 ± 0.04 ^d^	8.07 ± 0.07 ^c^	14.82 ± 0.03 ^c^	72.5	18.6	3.7
P-w	4.70 ± 0.04 ^c^	8.94 ± 0.08 ^b^	18.62 ± 0.03 ^c^	8.11 ± 0.06 ^c^	12.95 ± 0.04 ^d^	79.6	13.0	2.0
F-n	6.58 ± 0.01 ^a^	12.31 ± 0.03 ^a^	28.29 ± 0.06 ^b^	11.36 ± 0.01 ^a^	17.64 ± 0.01 ^b^	86.8	4.7	2.6
F-w	6.20 ± 0.07 ^b^	12.19 ± 0.07 ^a^	36.16 ± 0.06 ^a^	11.11 ± 0.01 ^b^	18.71 ± 0.06 ^a^	79.8	7.7	6.4

Data represent means ± standard deviations. For each column, values not displaying the same letter are significantly different (*p* < 0.05). d (0.1), d (0.5) and d (0.9) are the granule sizes wherein 10%, 50%, and 90% of all the granules by volume are smaller, respectively. D (3, 2) is the surface area-weighted mean diameter. D (4, 3) is the volume-weighted mean diameter. P1, P2, and P3 are the particle percentage of subgroups in different starches obtained by flow cytometry analysis.

**Table 3:** The weight-average molar mass (*Mw*), amylopectin chain length distribution, α-1,6 linkage percentage, and relative crystallinity of starches.

	*Mw* (×10^7^, g/mol)	Average Chain Length of Amylopectin (%)	Branching Degree (%)	Relative Crystallinity (%)	Chain Length Distribution (%)			
					DP 6–12	DP 13–24	DP 25–37	DP ≥37
P-n	2.4 ± 0.1 ^c^	20.1 ± 0.3 ^a^	2.8 ± 0.1 ^b^	37.6 ± 0.1 ^b^	18.0 ± 0.5 ^c^	45.4 ± 0.4 ^b^	17.8 ± 0.3 ^a^	9.1 ± 0.4 ^a^
P-w	17.0 ± 0.5 ^b^	19.2 ± 0.2 ^b^	4.2 ± 0.2 ^a^	38.4 ± 0.4 ^a^	20.8 ± 0.3 ^b^	47.4 ± 0.8 ^a^	14.1 ± 0.6 ^b^	7.7 ± 0.4 ^b^
F-n	2.8 ± 0.2 ^c^	20.2 ± 0.3 ^a^	2.8 ± 0.2 ^b^	34.1 ± 0.6 ^c^	18.5 ± 0.2 ^c^	44.4 ± 0.2 ^b^	18.1 ± 0.4 ^a^	9.4 ± 0.5 ^a^
F-w	22.3 ± 0.4 ^a^	17.9 ± 0.1 ^c^	3.9 ± 0.1 ^a^	36.5 ± 0.4 ^b^	28.1 ± 1.0 ^a^	44.0 ± 0.3 ^b^	13.7 ± 0.5 ^b^	5.4 ± 0.2 ^c^

Data represent means ± standard deviations. For each column, values not displaying the same letter are significantly different (*p* < 0.05). *Mw*, weight-average molar mass; DP, degree of polymerization.

**Table 4:** Pasting and thermal properties of starches.

Varieties	Pasting Properties							Thermal Properties			
	PV	TV	BD	FV	SB	PTM	PT	To (°C)	Tp (°C)	Tc (°C)	ΔH(J/g)
P-n	2110 ± 4 ^c^	997 ± 26 ^d^	1114 ± 30 ^d^	1275 ± 8 ^d^	1478 ± 4 ^b^	80.9 ± 0.0 ^a^	4.5 ± 0.0 ^a^	64.6 ± 0.5 ^c^	70.5 ± 0.1 ^c^	77.4 ± 0.1 ^c^	9.6 ± 0.2 ^b^
P-w	3286 ± 37 ^ab^	1097 ± 13 ^c^	2189 ± 24 ^a^	2575 ± 9 ^b^	279 ± 35 ^d^	77.8 ± 0.0 ^b^	4.2 ± 0.0 ^c^	71.1 ± 0.6 ^a^	77.9 ± 0.2 ^a^	82.3 ± 0.0 ^a^	10.8 ± 0.0 ^a^
F-n	3237 ± 16 ^b^	1348 ± 43 ^b^	1768 ± 6 ^c^	3599 ± 8 ^a^	2070 ± 8 ^a^	77.8 ± 0.0 ^b^	4.3 ± 0.0 ^b^	65.1 ± 0.3 ^c^	71.0 ± 0.4 ^c^	76.3 ± 0.5 ^d^	6.6 ± 0.2 ^d^
F-w	3299 ± 4 ^a^	1509 ± 28 ^a^	1890 ± 28 ^b^	1816 ± 7 ^c^	469 ± 36 ^c^	76.7 ± 0.0 ^c^	4.1 ± 0.1 ^c^	68.3 ± 0.3 ^b^	73.0 ± 0.3 ^b^	78.7 ± 0.1 ^b^	8.5 ± 0.4 ^c^

Data represent means ± standard deviations. For each column, values not displaying the same letter are significantly different (*p* < 0.05). PV, peak viscosity; TV, trough viscosity; BD, breakdown viscosity; FV, final viscosity; SB, setback viscosity; PTM, pasting temperature; PT, peak time. To, onset temperature; Tp, peak temperature; Tc, conclusion temperature; ΔH, gelatinization enthalpy.
